# Limbs Made to Measure

**DOI:** 10.1371/journal.pbio.1000421

**Published:** 2010-07-13

**Authors:** Anna Kicheva, James Briscoe

**Affiliations:** Medical Research Council (MRC) National Institute for Medical Research, Mill Hill, London, NW7 1AA, United Kingdom

*“Numerical precision is the very soul of science, and its attainment affords the best, perhaps the only criterion of the truth of theories and the correctness of experiments.” –D'Arcy Thompson*, On Growth and Form *(1917)*

This year marks the 150th anniversary of the birth of D'Arcy Thompson, the British biologist, classicist, and all round polymath (For more information on D'Arcy Thompson see www.darcythompson.org). Like many, he was fascinated by the appearance and structure of living matter, and in his influential book, *On Growth and Form* [1], he set out to describe and explain the principles of morphogenesis—the way living things grow and acquire their forms. Using a vast range of examples, from the honeycomb in beehives to the spirals in a snail's shell, he emphasized that form should be studied in the context of growth and that to explain shape it was essential to understand the underlying mechanisms. This led to the central thesis of the book: biological forms are the result of mechanical and physical processes that should be described with mathematical precision.

Yet, while the molecular basis of pattern formation and cellular differentiation during development has received much attention, our knowledge of the regulation of growth and organ shape lags behind. Partly, this is because acquiring accurate high-resolution 3-D measurements of organ shape and cellular behaviour and the quantitative analysis of these data has been technically challenging. Thus, 3-D organs are often studied using simpler 2-D representations. However, in recent years new imaging technology and the increase in computational power has begun to overcome these limitations, revealing previously unseen detail and allowing long-standing hypotheses to be tested.

In broad terms, the morphogenesis of a developing tissue is achieved by anisotropic growth. That is, the tissue expands in unequal amounts in different directions, so that the final organ shape gradually materializes. Two fundamentally different ways to achieve anisotropic growth can be envisioned (Figure 1). In one case, external mechanical forces mould the final organ form. As a result, cells are reshaped or rearranged by forces imposed from outside. For example, growth substrates exert surface tension on cultured cells, while blood flow exerts shear on endothelial cells (see [2]).

**Figure 1:**
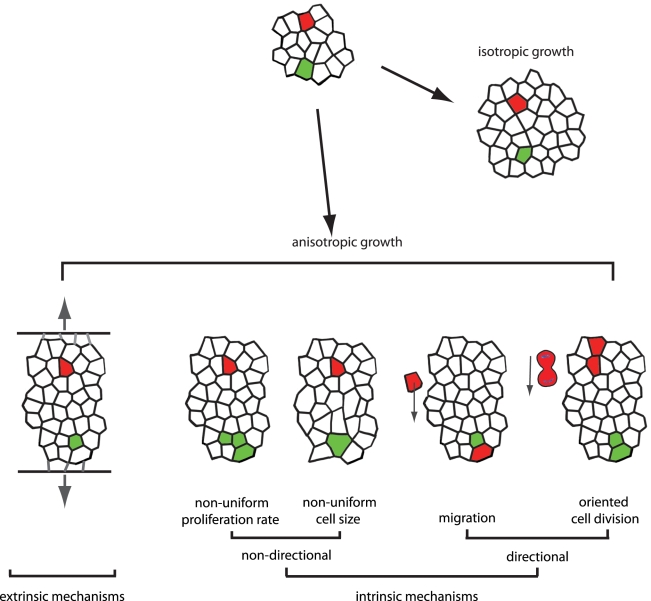
Examples of mechanisms that could account for changes in organ shape. In isotropic growth, the tissue grows equally in all directions. In anisotropic growth, there is more growth in some directions than others (in this case more growth occurs vertically, resulting in an elongated shape). Anisotropic growth can result from organ-extrinsic or intrinsic mechanisms. Extrinsic: the tissue elongates in response to directional forces imposed from outside. Intrinsic mechanisms result from two types of cell behavior: non-directional and directional. Non-directional: in non-uniform proliferation rate the orientation of divisions is random, but more divisions occur in some places in the tissue than others. Cell size can also change non-uniformly, resulting in different cell densities in different parts of the tissue. Directional: cell migration translocates cells in a preferred direction. Oriented cell division occurs when the spatial allocation of the daughter cells is directionally biased, resulting in elongated “clones” of cells. (The red and green cells are given for reference).

Alternatively, shape formation can be inherent to the organ and result from the collective behaviour of the individual cells comprising the organ. Importantly, two distinct classes of cellular behaviour can contribute to this active tissue modelling (Figure 1). In the first class, anisotropy results from cellular processes that occur non-directionally, but at different frequency across the tissue (e.g., proliferation, apoptosis, change of cell shape). For example, differences in proliferation rate across an organ could cause some parts to expand faster than others. For this to happen, cells must “know” only their position in a tissue, but not their spatial orientation. By contrast, the second class of mechanisms relies on directional—anisotropic—cellular activity. These could be, for instance, oriented division or migration of cells in a specified direction. Such mechanisms require a cue that provides cells with a bearing—a vector. Although fundamentally different, experimentally it has often proved difficult to distinguish between these classes of cell behaviour, since each can result in a cell changing its relative position within an organ. Moreover, these mechanisms are not mutually exclusive and a combination of passive, active, directional and non-directional cellular behaviours could play a role in defining organ shape. Thus, determining the contribution of different behaviour types is necessary for understanding the molecular mechanisms of organ morphogenesis.

One tissue that exemplifies the problem of distinguishing the mechanism of morphogenesis is the developing limb. From amphibians to mammals, the limbs of tetrapods start growing from small bulges called limb buds. Initially, these buds are composed of loose mesenchymal cells, ensheathed by a layer of ectodermal cells (Figure 2). At the distal rim of the limb bud, the ectoderm is thickened into the “apical ectodermal ridge” (AER), which secretes extracellular signals, notably members of the Fibroblast Growth Factor (FGF) family, that are important for limb outgrowth and patterning (see [3]). Following limb bud initiation, but prior to the laying down of the skeletal elements, limb tissue extends mainly in a distal direction, away from the body, such that the length along the proximal–distal axis increases much faster than the anterior–posterior or dorsal–ventral axes. Thus, the developing limb serves as a good example of anisotropic growth and raises the question of what mechanisms contribute to the distal outgrowth.

**Figure 2:**
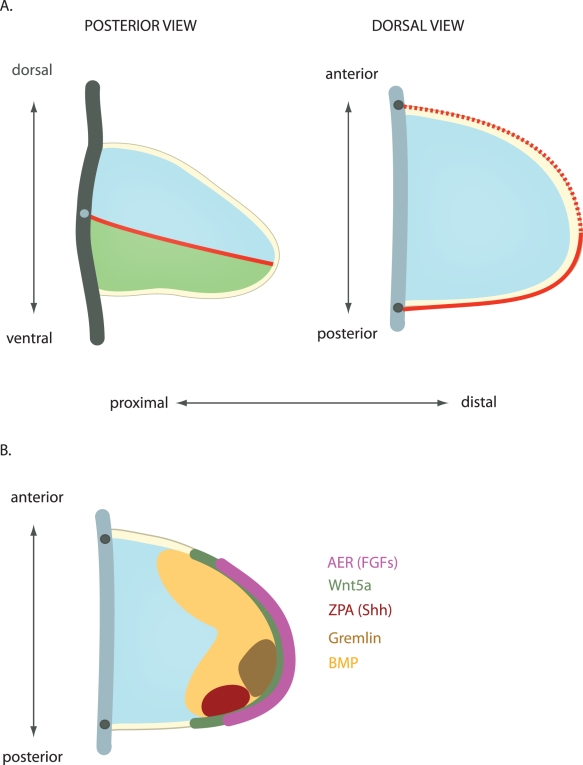
Geometry and patterning of the limb bud. A) Geometry of the limb bud. Yellow - ectodermal layer. Blue – dorsal. Green - ventral mesenchyme. Red line indicates the dorsoventral boundary (solid - posterior, dashed - anterior). The thick grey lines represent the flank, with the dots indicating the points of cross-section between the posterior and the dorsal view. B) Expression domains of patterning signals. The AER - apical ectodermal ridge, expresses FGF encoding genes (*Fgf8*, *Fgf4*, *Fgf9*, *Fgf17*). The ZPA-zone of polarizing activity, is the source of Sonic Hedgehog (Shh). Bone morphogenetic protein (BMP4) is expressed in a broad domain, which is progressively restricted in time. Gremlin1 is a BMP antagonist. BMP, Gremlin, Shh, and FGF are interlinked in signaling feedback loops, which causes their domains of expression and activity to change over time (see [37]). Wnt5a is expressed in a proximo-distal gradient, with highest levels at the tip of the limb bud [38].

The realization that the AER is the source of a proliferative signal has provided the inspiration for a “growth-based morphogenesis” model of limb development [4],[5]. According to this view, proximal–distal elongation of the limb bud results from a gradient of proliferation rates along this axis, which represents a non-directional mechanism. Indeed, measurements of cell cycle duration confirmed that distally located cells proliferate faster [6]. Moreover, computational models, some dating back more than 40 years, have been used to check if these differences in proliferation rate could explain limb morphogenesis. These models were restricted to one- or two-dimensional representations of the limb and suggested that “growth-based morphogenesis” could be responsible for shaping the limb. But, other mechanisms were not ruled out, and it is notable that in some models directional behaviors or external mechanical constraints were included to make them conform more closely to empirical observation [7]–[11].

In an article in this issue of *PLoS Biology*, Boehm et al. revisit the “growth-based morphogenesis” model, utilizing the latest imaging and computation techniques. Their approach differs in three significant ways from previous studies and, in the spirit of D'Arcy Thompson, offers a new level of precision. First, Boehm et al. used the recently introduced technique of Optical Projection Tomography (OPT) [12] to produce a 3-D high-resolution image of the growing mouse limb. With this they generated an in silico limb bud that can be used to test any model of limb morphogenesis. Second, Boehm et al. systematically collected proliferation rate and cell density data, which, unlike previous studies, do not depend on assumptions about the length of cell cycle phases. These data were combined to produce a 3-D map of proliferation rates in the developing limb bud. Finally, the authors constructed a 3-D computer model to simulate how the realistic OPT replica would grow given the measured proliferation rates. In this model, the volume of the limb was subdivided into ∼27,000 connected tetrahedral building blocks, called “finite elements.” The effect of growth was simulated by increasing the volume of each finite element at a rate corresponding to the proliferation rate at that location in the limb. To determine how the simultaneous expansion of all the elements and their influence on each other affected overall limb shape, the authors used the observation that the limb mesenchyme has physical properties similar to an incompressible viscous fluid. This allowed them to use principles from fluid mechanics to predict the expansion and trajectory of each element from the measurements of proliferation rate. The model was tested by comparing the simulated to the real limb bud shape.

What does this analysis tell us? First, the data rule out “growth-based morphogenesis” as the main driver of morphogenesis. Although Boehm et al. confirm that distal proliferation rates are twice those of proximally located cells, these differences cannot account for the resulting limb shape. Second, using the computer model to systematically explore a wide range of growth rates, the authors show that it is theoretically possible to produce the observed limb shape using non-directional mechanisms. For this to happen, however, some regions would have to have cell cycle times of less than 2h—at least 5 times faster than observed—whereas in other regions, up to 10% of the cells would have to shrink or die. The modelling also indicated that a significant number of cells (∼20%) enter the limb bud from the flanking mesenchyme during this period of morphogenesis. One possibility, therefore, is that the cells entering the limb push those already in the limb bud in a distal direction. However, Boehm et al. argue that the evidence does not favour this mechanism. Instead, they find a directional bias to the filopodia extensions and to the orientation of division of cells throughout the limb bud mesenchyme. This indicates that some kind of active directional—anisotropic—cell behaviour is the most likely explanation for the changes of limb shape.

The study refocuses attention on active directional mechanisms of morphogenesis. Similar conclusions have been reached in studies of 2-D tissues, such as epithelial sheets. Most notably, biases in the orientation of cell division are involved in shaping both the *Drosophila* wing disc and the petals of flowering plants [13]–[15], suggesting that this mechanism might be commonly used in development. Future studies will need to address how directional cell behaviours account for limb growth. Boehm et al.'s data suggest it might involve a convergence-extension–like process, rather than straightforward distal movement. Addressing this issue will require tracing the trajectories of individual cells, using in vivo imaging techniques. This is technically challenging because of the size and opacity of limb buds and the difficultly in culturing the tissue in a suitable way for time-lapse imaging. However, improvements in automated cell tracking and techniques, such as live OPT and fluorescence light-sheet microscopy, are making promising advances in this direction [16]–[18]. In addition, to interpret such imaging data, it will be necessary to develop methods for 3-D data analysis and biophysical mechanical models of the cellular behaviours and forces that contribute to the emergent anisotropic tissue growth.

Another major question that arises from these studies is the nature of the cues responsible for anisotropic cell behaviours. Such cues could be biochemical or mechanical. Several secreted signals form gradients in the limb bud and regulate the growth and patterning of the tissue (Figure 2) [3]. Moreover, these gradients were initially proposed to provide the tissue with some inherent polarity [19]. But direct involvement of morphogens in anisotropic cell behaviours, such as oriented division or migration, has received only limited attention recently [14],[20]. In the limb, one study showed that an ectopic FGF4 source causes displacement of mesenchymal cells towards it [21], thus raising the possibility that FGF emanating from the AER regulates directional behaviour. This would be consistent with the role of FGF signaling in guiding cell migration during gastrulation [22],[23]. In addition, it might be significant that the shortened and widened limb shape of Talpid3 mutant chick embryos looks similar to Boehm et al.'s computer predictions of limb buds lacking directional cell movements. The Talpid3 gene encodes a centrosomal protein involved in forming cilia [24]. As a consequence Sonic Hedgehog (Shh) signaling, which is required for patterning the anterior–posterior axis of the limb bud, is defective in Talpid3 mutants [25]. However, the motility and adhesion of isolated Talpid3 mutant mesenchymal limb bud cells is also abnormal [21]. Whether this motility defect is related to Shh signaling, or to a different role of cilia, such as mechanosensing [26], and whether it contributes to the abnormal limb shape remains to be investigated.

In addition, cells could also acquire a sense of direction in response to the planar cell polarity (PCP) pathway. PCP signaling is involved in reorganizing epithelial packing geometries (e.g., during *Drosophila* wing development [27]) and in convergent extension movements [28]. PCP has been shown to contribute to organ shape via controlling the directional bias of cell activities, such as cell elongation, junction remodeling, or orientation of the division axis [29]. These anisotropic processes are accompanied by changes in the mechanical forces exerted by cells on their neighbours, and could be mediated via junctional or cytoskeletal components (e.g., [30],[31]). However, the precise molecular mechanisms and function of the pathway are not fully understood [32],[33]. The involvement of PCP in vertebrate limb development has not been explored, but mutants lacking Wnt5a, a PCP regulator, have shortened limbs [34],[35]. Whether this is because of a role for planar polarity in the directional behaviour of limb cells is not clear. Thus, it remains to be determined to what extent cells' “sense of direction” emerges from local mechanical forces, or depends on initial asymmetries in tissue structure and boundaries, or on global external cues.

In conclusion, directional cell activities, such as oriented division or migration, appear to play a key role in organ morphogenesis. However, the cues and forces that provide cells with an orientation vector to achieve this anisotropic cell behaviour remain to be fully explored. Future studies need to identify which processes are directional, how these contribute to organ shape, and how they are coordinated with pattern specification and growth. This highlights the need for a systems approach providing an integrative understanding of different processes that are concurrent during organogenesis (also see [36]). And almost 100 years after D'Arcy Thompson pointed this out, we are reminded that the study of morphogenesis requires knowledge of the relationship between growth and form, acquired from precise experimental observations and interpreted in the context of biophysical laws.

## Abbreviations

AER: apical ectodermal ridge
OPT: Optical Projection Tomography
PCP: planar cell polarity
